# Deep mutational scanning reveals transmembrane features governing surface expression of the B cell antigen receptor

**DOI:** 10.3389/fimmu.2024.1426795

**Published:** 2024-07-23

**Authors:** Samyuktha Ramesh, Margareta Go, Matthew E. Call, Melissa J. Call

**Affiliations:** ^1^ Structural Biology Division, Walter and Eliza Hall Institute of Medical Research, Parkville, VIC, Australia; ^2^ Department of Medical Biology, University of Melbourne, Parkville, VIC, Australia

**Keywords:** B cell receptor (BCR), receptor assembly, deep mutational scanning, transmembrane domains, receptor expression

## Abstract

B cells surveil the body for foreign matter using their surface-expressed B cell antigen receptor (BCR), a tetrameric complex comprising a membrane-tethered antibody (mIg) that binds antigens and a signaling dimer (CD79AB) that conveys this interaction to the B cell. Recent cryogenic electron microscopy (cryo-EM) structures of IgM and IgG isotype BCRs provide the first complete views of their architecture, revealing that the largest interaction surfaces between the mIg and CD79AB are in their transmembrane domains (TMDs). These structures support decades of biochemical work interrogating the requirements for assembly of a functional BCR and provide the basis for explaining the effects of mutations. Here we report a focused saturating mutagenesis to comprehensively characterize the nature of the interactions in the mIg TMD that are required for BCR surface expression. We examined the effects of 600 single-amino-acid changes simultaneously in a pooled competition assay and quantified their effects by next-generation sequencing. Our deep mutational scanning results reflect a feature-rich TMD sequence, with some positions completely intolerant to mutation and others requiring specific biochemical properties such as charge, polarity or hydrophobicity, emphasizing the high value of saturating mutagenesis over, for example, alanine scanning. The data agree closely with published mutagenesis and the cryo-EM structures, while also highlighting several positions and surfaces that have not previously been characterized or have effects that are difficult to rationalize purely based on structure. This unbiased and complete mutagenesis dataset serves as a reference and framework for informed hypothesis testing, design of therapeutics to regulate BCR surface expression and to annotate patient mutations.

## Introduction

The B cell antigen receptor (BCR) is a tetrameric glycoprotein complex (1, 2) on the surface of B lymphocytes responsible for recognizing foreign matter in the body and communicating this information into the B cell. It is composed of the membrane-bound immunoglobulin (mIg) light chain (LC) and heavy chain (HC), which together form a transmembrane domain (TMD)-tethered antibody that binds foreign antigens (3), and the CD79AB heterodimer, which provides the cytoplasmic tails with immunoreceptor tyrosine based activation motifs (ITAMS) (4) to dock the intracellular signaling apparatus. Signaling through the BCR complex is required for normal B cell development and for activation of high-quality antibody production during immune responses. Failure to express the BCR on the cell surface results in B cell pathologies characterized by developmental defects and impaired immune function (5). Unregulated BCR signaling, on the other hand, contributes to the development and persistence of B cell-derived cancers (6, 7), as evidenced by the effectiveness of inhibitors targeting the proximal signaling proteins Bruton tyrosine kinase (BTK) and PI3K in B-cell leukemias and lymphomas (8).

**Figure 1:**
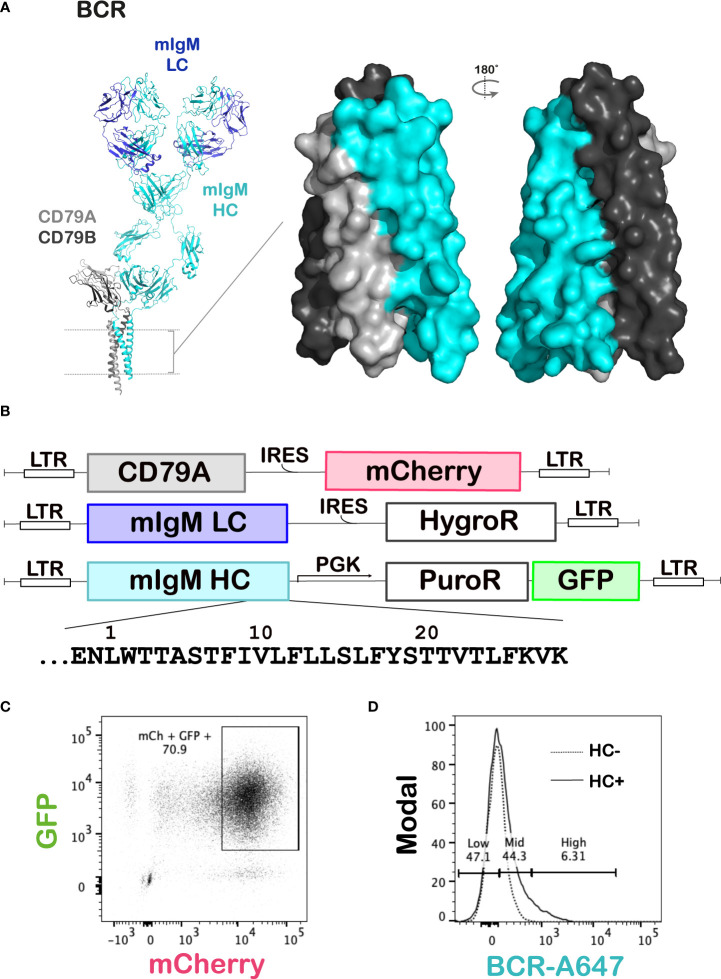
Deep mutational scanning of the BCR mIgM HC TMD. **(A)** Left: The cryo-EM structure of the mouse IgM BCR (PDB ID: 8EMA) showing its components – the antigen binding mIg and the signaling CD79AB dimer. The dotted lines represent the cell membrane. Right: Surface rendering of the BCR TMD showing the extensive contact between the mIg and CD79AB in this region. **(B)** Schematics of the expression constructs for each gene transduced into the J558L mouse B cell line. The amino acid sequence of the region mutated by DMS in the HC TMD and juxtamembrane region is shown under its construct map. LTR: long terminal repeat, IRES: internal ribosomal entry site, HygroR: hygromycin resistance gene, PGK: phosphoglycerate kinase 1 promoter, PuroR: puromycin resistance gene, GFP: green fluorescent protein. **(C, D)** Gating strategy for the BCR DMS assay. The CD79A and mIgM LC constructs shown in **(B)** were transduced into J558L cells and selected by flow cytometry and with hygromycin, respectively, to generate the recipient line for the DMS library. The mIgM HC DMS library was then transduced into this recipient line and transductants selected with puromycin. The cells were then surface stained with an anti-IgM antibody. Live cells, followed by GFP and mCherry double-positive cells were gated first **(C)**, and this population was then gated on the level of staining of surface IgM **(D)**. The dotted line represents the background staining of the recipient line with no transduced HC, and the solid line represents staining of the cells transduced with the HC DMS library at low MOI.

Like antibodies, BCRs exist in five isotypes – IgM, IgD, IgG, IgA and IgE – with distinct structures and functionalities (9). In each case, the isotype-specific mIg and the invariable CD79AB must co-assemble in the endoplasmic reticulum (ER) before a functional BCR can be transported to the cell surface (10). Several interactions required for this assembly occur through highly conserved polar residues in the TMDs of these subunits (11), as evidenced by substantial biochemical work showing that the complex is sensitive to mutations in the mIg TMD (12–20). Recently, this has been further corroborated by cryogenic electron microscopy (cryo-EM) structures (21–23) revealing the large packing surfaces between mIg and CD79AB in the TMD (**Figure 1A**), which account for ~85% of the total buried surface area between these subunits within the complex. These structures also highlight various other potential interactions contributing to BCR assembly that appear to be mediated by hydrogen bonding, complementary hydrophobic packing and van der Waals interactions.

To understand the precise nature of the interactions holding the BCR complex together and their relative importance, we performed deep mutational scanning (DMS) on the mouse mIgM heavy chain TMD, mutating each native residue to every other amino acid and measuring their effects on surface expression in a single pooled assay. With these 600 single-amino-acid variant proteins, we dissect which biochemical interactions contribute to stabilizing the mIg dimer versus assembly with CD79AB, and compare the results with previously reported mutagenesis, disulfide crosslinking, computational modeling and biophysical data.

## Methods

### Genes and plasmids

The CD79A coding sequence was obtained from mouse splenic B cell total mRNA (kindly provided by Phil Hodgkin, WEHI, Melbourne, Australia) that was reverse-transcribed to cDNA and then amplified by PCR with primers specific to the native CD79A sequence. The PCR fragment was cloned into a retroviral pMSCV-IRES-mCherry backbone by restriction ligation. HyHEL10 (high affinity monoclonal, specific for hen egg lysozyme) heavy chain (HC) and kappa light chain (LC) genes were ordered as gene blocks from Integrated DNA Technologies (IDT, Coralville, USA). AttB fragments were added to the ends of the LC by PCR to clone the fragment into the Gateway cloning vector pDONR221 (Thermo Fisher Scientific, Waltham, USA) using BP clonase (Invitrogen, Waltham, USA). This was used as the entry clone for LR cloning (Invitrogen, Waltham, USA) into the gateway expression vector pMX-IRES-HygroR (kindly provided by Andrew Brooks, University of Queensland, Brisbane, Australia). An AttL2 fragment was added to the end of the HC sequence by PCR and then the PCR fragment was cloned into the Gateway donor vector pDONR221 (Thermo Fisher Scientific, Waltham, USA) with MPL signal sequence by restriction ligation. The pDONR construct was then used as the entry clone for LR cloning (Invitrogen, Waltham, USA) into pMX-GW-PGK-PuroR-GFP (24). Virus packaging vectors used were MMLV-gag-pol and VSVg (retroviral, for CD79A and HC) or pRSV-Rev, pMDLg/pRRE and VSVg (lentiviral, for LC).

### DMS library construction

PCR fragments containing the HC TMD mutations were generated using the wildtype HC pDONR plasmid described above as template and forward primers with randomized codons (NNN) at each position to be mutated separately (30 positions, numbered E-2 to K28, where position 1 is assigned to the first predicted TMD residue (25)) and an invariant reverse primer. These mutation-containing PCR fragments were then combined with the vector backbone fragment (also generated by PCR) by HiFi assembly (NEB, Ipswich, USA). Assembly products were transformed into NEB^®^ 10-beta Electrocompetent *E.coli* cells and the resultant colonies pooled for plasmid isolation. This plasmid library on the pDONR backbone was cloned with LR clonase into the pMX-GW-PGK-PuroR-GFP backbone vector described above to generate the final library. This library was checked by Illumina sequencing to ensure coverage of all possible amino acid variants.

### Cell lines

The mouse B cell line J558L in which the DMS assay was performed was obtained from the Corcoran Lab, WEHI, Melbourne, Australia. The cells were cultured in RPMI (Gibco, Thermo Fisher Scientific, Waltham, USA) with 10% FBS (Gibco, Thermo Fisher Scientific, Waltham, USA) and 2 mM L-Glutamine (Gibco, Thermo Fisher Scientific, Waltham, USA). Human embryonic kidney (HEK) 293T cells (Cellbank Australia) used for virus production were cultured in DMEM (Gibco, Thermo Fisher Scientific, Waltham, USA) with 10% FBS and 2 mM L-Glutamine.

### Transfection and transduction

The target genes were transfected with the packaging vectors described above into HEK 293T cells using calcium phosphate precipitation for virus production. The viral supernatant was harvested after 48 hours and added to 1 x 10^6^ J558L cells. Polybrene (Sigma-Aldrich, Burlington, USA) was added at 8 ug/ml and the cells were transduced by centrifugation at 1000 g for 45 minutes at 32 °C. For the HC DMS library, three independent retroviral transductions were performed and treated as separate replicates for all subsequent steps. Transduction efficiency was checked after 48 hours by flow cytometry and transduced cells were enriched by fluorescence activated cell sorting (for CD79A) or selection on 25 ug/ml puromycin (Thermo Fisher Scientific, Waltham, USA) (for the HC) or 600 ug/ml hygromycin (Merck, Darmstadt, Germany) (for the LC) for 45-72 hours. Following antibiotic selection, live cells were separated from dead cells by Ficoll separation (Sigma-Aldrich, Burlington, USA).

### Surface expression DMS assay

Four days post-transduction, J558L cells were stained with Alexa Fluor647 anti-mouse IgM (Cat. No. 406526, BioLegend, San Diego, USA) at 2.5 µg/ml per 2 x 10^5^ cells per 50 µL staining volume on ice for 30 minutes, then sorted by flow cytometry. The cells were gated as per **Figure 1D**, with the positive peak tail in the High gate and the negative peak split into equal halves as the Mid and Low gates. After the sort, the cells from each population were expanded for four days, until each sample had at least 5 x 10^5^ cells, then mRNA was extracted from 5 x 10^5^ cells from each sample using the RNeasy kit (Qiagen, Hilden, Germany).

### Illumina sequencing

From 1 µg of the extracted mRNA, cDNA was prepared for each replicate using a reverse primer that annealed 3’ to the mutated region of the HC and containing a 16 bp unique molecular identifier (UMI) and an Illumina adapter for subsequent amplification. Illumina amplicons were generated using a forward primer that annealed 5’ to the mutated region and a reverse Illumina adapter primer. This 184 bp product was then multiplexed with indexing primers in triplicate. Samples were paired-end sequenced using an Illumina NextSeq kit (P1 Nextseq 2000, 300 cycles), with 162 cycles in the forward direction and 176 cycles in the reverse direction.

### Data analysis

The paired-end reads were de-multiplexed using Cutadapt (version 4) (26) and forward and reverse reads were merged with USEARCH (v8.1.1861_i86linux32) (27). Around 5 x 10^5^ reads were obtained per sample. Adapter sequences were removed using Cutadapt, and UMIs deduplicated using UMI-tools (version 1.1.4) (28) and samtools (29). After comparing the data from the Low, Mid and High gates in various combinations, we determined that the best representation resulted from comparing the data from the Mid and High gates combined against the data in the Low gate. This is likely due to the relatively small shift seen in the surface staining of the BCR DMS library (**Figure 1D**). Using seqkit (version 2.8) (30), reads from the High and Mid populations were combined for each of the three replicates by first equalizing the total reads, then scaling based on the cell numbers obtained for each population after the flow cytometry sort. Variant fitness scores and error estimates were calculated for this combined Mid/High population compared to the Low population using DiMSum (version 1.3) (31). Good agreement was seen between the variant fitness scores from the three replicates (**Supplementary Figure 2**). Since DiMSum fitness scores are normalized to the wildtype sequence, which is overrepresented in the library, we rescaled the fitness scores and errors between the weighted mean fitness and error of the synonymous wildtype variants (*F_mean(synWT)_*, set to 1) and the weighted mean fitness and error of the premature stop codons (*F_mean(STOP)_*, set to 0). The new variant fitness scores (*F_new_*) and errors (*Sigma_new_*) were calculated using the following equations:


$$F_{new}\;=\;k*F_{old}\;+\;b$$

$$Sigma_{new}\;=\;k*Sigma_{old}$$

$$\text{where }k,\text{ the scaling factor }=\frac{1}{\left(F_{mean(synWT)}\;-\;F_{mean\left(STOP\right)}\right)}$$

$$\text{and }b,\text{ the translation constant }=\;1-\;k\;*\;F_{mean\left(synWT\right)}$$

## Results

### DMS library and selection strategy

Surface expression of a mouse IgM BCR was reconstituted in the J558L mouse B cell line, which expresses CD79B but lacks CD79A and mIg HC. Constructs encoding mouse CD79A, HyHEL10 (32) IgM HC and kappa LC were virally transduced into the cells (**Figures 1B, C**). First, a recipient line was generated by transduction of CD79A and LC into the J558L cells, followed by selection of transductants on hygromycin and flow-sorting for the top 10% mCherry (CD79A)-expressing cells. Wildtype HC was then transduced into this recipient line, and BCRs that had assembled and translocated to the cell surface were stained with an anti-IgM monoclonal antibody conjugated to A647 fluorophore. The BCR was successfully expressed at the cell surface when all three of these subunits were present (**Supplementary Figure 1**).

To thoroughly interrogate the TMD interactions required for BCR surface expression, single amino acid substitutions were made in the transmembrane and juxtamembrane sequences of the mIgM HC [30 positions numbered E-2 to K28, where position 1 is assigned to the first predicted TMD residue (25)] using fully degenerate codons to replace the native residue with every alternative amino acid and premature stop codons. A library of plasmids containing these variant HCs was generated (see Methods), then packaged into retrovirus particles and transduced in three independent replicates (**Supplementary Figure 2**) into the J558L recipient line expressing CD79A and LC as described above, ensuring a multiplicity of infection (MOI) of approximately 0.1 (transduction efficiency ~10%). Transductants were selected on puromycin and sorted on BCR surface expression by flow cytometry (gating strategy shown in **Figures 1C, D**). The sorted cell populations were then expanded in culture, and their mRNA was extracted for reverse transcription and Illumina sequencing of the mutated region of the mIg HC. The sequencing results provided the relative frequencies of all variants in each sorted population, and these values were used to compare the degree of enrichment in the surface BCR-High and -Mid gates (combined) versus the surface BCR-Low gate and calculate fitness scores and errors in the DiMSum software package (see Methods) (31).

### BCR surface expression sequence-function heatmap

**Figure 2:**
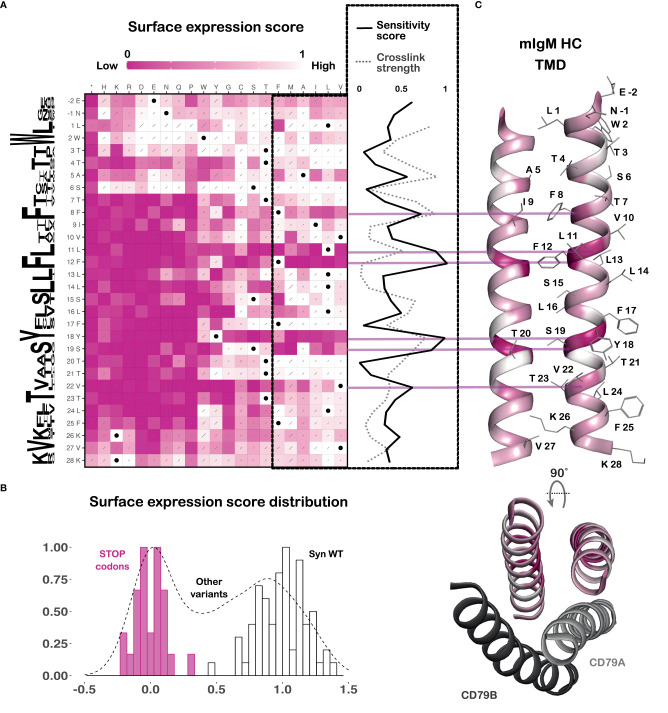
BCR surface expression DMS reveals a feature-rich TMD. **(A)** Sequence-function heatmap of the BCR surface expression DMS results, normalized between wildtype-level surface expression (white, set to 1) and premature terminations (pink, set to 0). Black dots indicate wildtype sequence and grey slashes represent error estimates. A sequence conservation Weblogo (33) generated using sequences of the five human and mouse BCR isotypes is shown along the left of the heatmap. A sensitivity score (normalized between 0 and 1, where 0 = least sensitive and 1 = most sensitive) for each TMD position was calculated by averaging the scores of mutations to all amino acids that are energetically favorable in a TMD (boxed in dashed black lines). This is plotted to the right of the heatmap as a solid black line. Results from previously reported cysteine crosslinking experiments (20) (normalized between 0 and 1, where 0 = no crosslinks and 1 = strong crosslinks) are plotted as a grey dashed line on the same scale to demonstrate their close concordance. Data are from three independent retroviral transductions of the HC DMS library. **(B)** The distribution of the DMS data, with the scores of the synonymous wildtype variants in white bars, the early termination mutants in pink bars, and all other mutants in the black dashed histogram. **(C)** Top: Cartoon representation of the mouse mIgM HC TMD (PDB ID: 8EMA) colored by the sensitivity score for each position. Amino acid side chains are shown as grey lines. Bottom: Top-down view of the above HC TMD showing the relative positions of the CD79A and B TMDs.

The fitness scores are shown as a sequence-function heatmap (**Figure 2A**), where white variants were enriched in the surface BCR-positive population and pink variants were enriched in the surface BCR-negative population. For ease of interpretation, the enrichment scale is normalized between the scores of synonymous wildtype variants (white, successful surface expression, score set to 1) and those of premature stop codon mutants (pink, complete lack of surface expression, score set to 0) (**Figure 2A**). Histograms show the distribution of the scores of the synonymous wildtype variants, premature stop mutants and the rest of the library (**Figure 2B**). The clear separation between the peaks for synonymous wildtype and premature stop codons confirms that the assay successfully distinguishes between BCR variants that are and are not at the cell surface, respectively. A sequence conservation Weblogo (33) is included along the left of the heatmap to show the evolutionary conservation of each residue across all five human and mouse isotypes. A sensitivity score has also been calculated for each position, averaging the scores of all variants where the native amino acid has been changed to any of the most energetically favorable TMD residues (shown in the dashed box) (34, 35) as a measure of how specific the structural requirements are at each position. This score has also been depicted on the structure of the mIg dimer (**Figure 2C**), highlighting the faces of the helices that most strongly impact surface expression.

To ensure that the sort for cell-surface expression was the only selection pressure at play in our screen, and not, for example, population drift due to growth advantages or disadvantages conferred by particular variants, we compared the raw fitness scores of the recombined data from all sorted populations (representing unselected cells) with the plasmid DNA library used to generate retrovirus (representing the starting variant distribution; see **Supplementary Figure 3**). We observed no substantial or position-specific differences in variant frequencies from this comparison, and synonymous wildtype and premature stop variants did not segregate, indicating that starting and ending library contents are very similar when the selection step (flow sorting) is taken out of the analysis. We can therefore confidently interpret the heatmap in **Figure 2A** as an accurate reflection of the effects of each substitution on BCR surface expression.

### General properties of the TMD

The heatmap demarcates the boundaries of the strictly membrane-embedded sequence as T7 to F25, reflected in the signature of near-complete intolerance to substitution with charged amino acids or proline that breaks down above and below this (**Figure 2A**). The cryo-EM structure indicates the helical structure is maintained for two additional turns above T7 and one additional turn below F25 (21), but as these positions display a weaker and non-contiguous TMD signature, they may be dynamically or only partially membrane-embedded. The computational TMD prediction tools TMHMM-2.0 (36) and DeepTMHMM (25) predict the membrane spanning regions to be T3-F25 and L1-V23, respectively. In the absence of intact BCR structures in lipid bilayers, we suggest that the contiguous intolerance to charged, strongly polar and proline residues from T7 to F25 (19 amino acids) provides the most accurate experimental definition of the mIg TMD.

Within this strictly membrane-embedded region, most positions allow for mutation to other uncharged residues, except at some key positions that are highly sensitive to mutation. These include F8, L11, F12, Y18 and V22. Maintaining helicity in this region is strongly preferred, with the potentially rigid, destabilizing kinks introduced by proline being detrimental and the flexibility of glycine being tolerated only in one small stretch (F17 and S19-T21). As expected, premature stop codons abrogate surface expression, including at the first two intracellular tail positions (K26, V27), which are likely required for proper membrane anchoring. On either side of the TMD, the documented positive-inside (37) and negative-outside (38) trends are largely maintained, with R and K generally unfavorable at the N terminal juxtamembrane region and E and D generally unfavorable at the C terminal end.

### Most immutable residues lie at the mIg dimer interface

Previously reported cysteine crosslinking experiments in the mIg TMD highlighted a clear dimerization interface that is a crucial determinant of BCR stability (19, 20). Overlaying the crosslinking strength at each position of the mIg TMD from that study with the sensitivity score calculated here shows the two are largely correlated (**Figure 2A**). Positions that are least tolerant to mutation showed the highest propensity to crosslink and lie at the mIg dimer interface: A5, F8, L11 and Y18 (**Figures 2A, C**). Conversely, this plot also highlights position F12 that is crucial to BCR stability, but not by virtue of contributing significantly to the mIg dimer interface because it has a very low propensity to crosslink. Rather, it interacts with CD79AB as discussed below.

### Features that are critically required for surface expression

**Figure 3:**
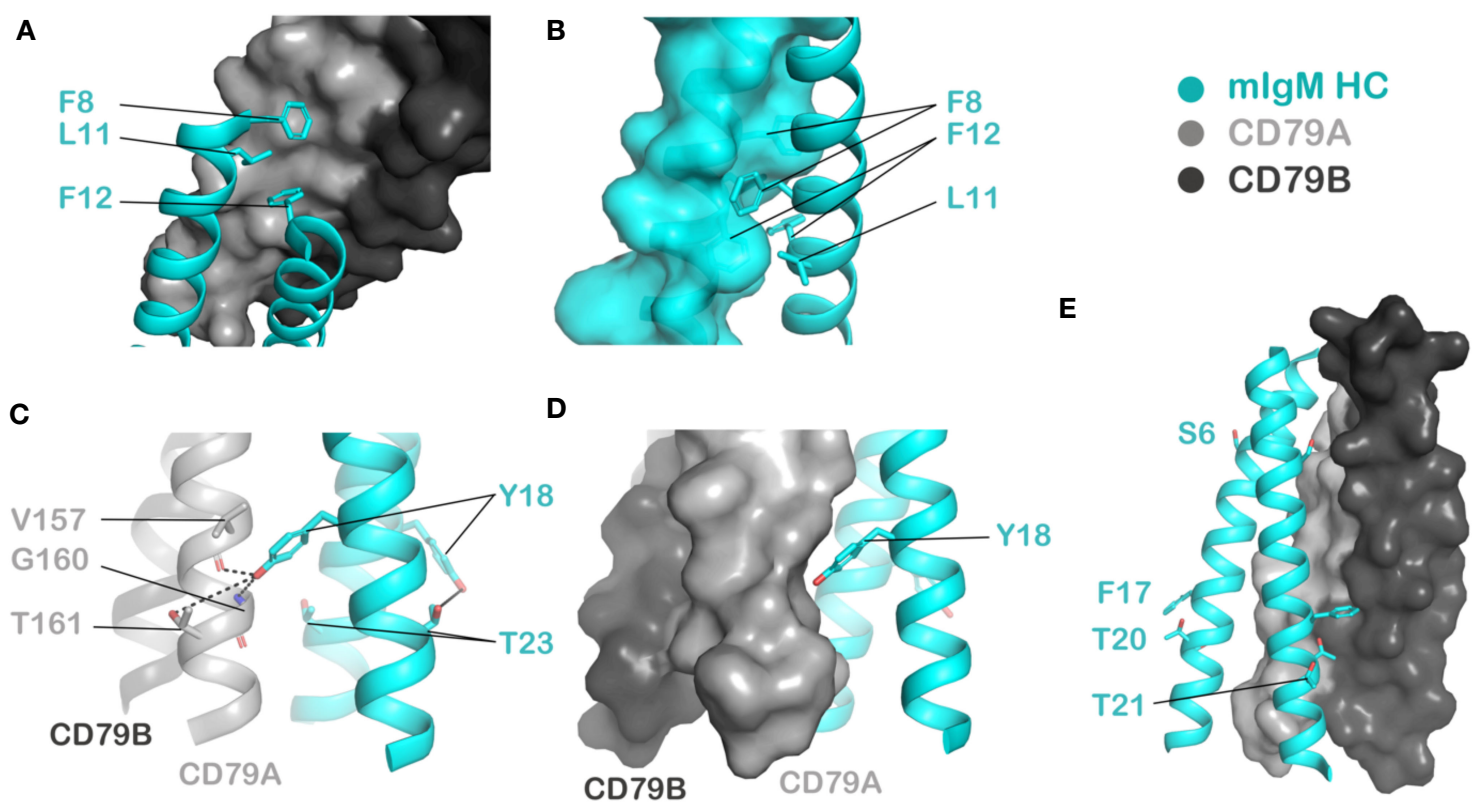
Visualizing noteworthy amino acid positions highlighted by the DMS results in the context of the mouse IgM BCR cryo-EM structure (PDB ID: 8EMA). HC: cyan, CD79A: light grey, CD79B: dark grey. **(A, B)** Highly immutable residues F8, L11 and F12 fit into complementary grooves in the surface of CD79A **(A)** as well as the partner HC protomer **(B)**. **(C)** Y18 is proposed to be involved in polar interactions with the partner HC protomer’s T23 on one side of the HC dimer and with V157, G160 or T161 in CD79A on the other side. **(D)** Aromaticity at Y18 may be required for complementary packing against CD79A. **(E)** The residues least sensitive to mutation - S6, F17, T20 and T21, face the membrane interior on one HC protomer and are adjacent to CD79B on the other protomer.

Hydrophobicity is required at various positions including L11 and V22, with L11 intolerant of mutation to any other amino acid. V22, which has not previously been highlighted as a driver of any key interactions but is evolutionarily conserved (**Figure 2A**), displays less stringent requirements but strongly favors branched aliphatic amino acids I/L/V. At F8 and F12, aromaticity is important as mutation to anything but Y results in a marked reduction in surface expression. The cryo-EM structure indicates that these residues are involved in complementary packing between the two mIg chains as well as with CD79A (**Figures 3A, B**), though the more severe effects of mutations at F12 than at F8 in our data suggest that F12 makes a more important energetic contribution.

As for the polar residues previously reported to be involved in intra-BCR interactions, Y18 is largely intolerant to mutations except to phenylalanine and tryptophan, suggesting both aromaticity and polarity are important here. Consistent with this, the cryo-EM structures place Y18 to interact via intermolecular hydrogen bonds with several possible partners including a threonine side chain (T161) (23), a backbone amide (G160) (21) or and backbone carbonyl (V157) (22) in CD79A (**Figure 3C**). Our previous mutagenesis and modelling also showed that the Y18 on the side facing away from CD79AB interacts with T23 in the partner mIg TMD to form a stabilizing Y-T hydrogen bond staple (19, 20) (**Figure 3C**). The preference for aromaticity at Y18 is likely related to complementary packing with CD79 TMDs (**Figure 3D**).

The neighboring S19 tolerates mutation to other small amino acids A/C/G, while all larger amino acids I/L/V/M/F/Y/W are disruptive, indicating that close packing is critically required here. This is consistent with results from previous site-directed mutagenesis performed on BCRs assembled in ER microsomes (19, 20), as well as studies showing the mutant S19A has no functional defects (13, 14). There is also evolutionary conservation of a small amino acid at this position for all proteins containing the interchain Y-T hydrogen bond staple described above (19, 20), likely for the purpose of accommodating close helix-helix packing. Another small polar interface residue, S15 one turn above, is less sensitive to mutation than S19, but cannot be replaced with bulky aromatic residues F, Y or W. This is also in agreement with our previous BCR assembly experiments in ER microsomes showing S15L is tolerated (19, 20). However, these experiments also found mutations that prevent hydrogen bonding like S15A and S15V destabilized the complex in detergent extracts of ER membranes, and this apparently does not translate to reduced surface expression in the DMS assay.

### Positions that are least sensitive to mutation

The positions most tolerant to mutation in the TMD are S6, F17, T20 and T21, which all lie on the same face of a helix (**Figure 3E**). These are among the least conserved positions in mIg TMDs across species/isotypes (**Figure 2A**), and they all face outward into the lipid bilayer on one protomer in the mIg dimer. On the other protomer, S6, F17 and T20 are on surfaces that face CD79B, but they do not appear to form crucial contacts: F17 is peripherally situated where any other hydrophobic amino acid should be accommodated, while S6 and T20 have no intermolecular hydrogen bonding partners available (**Figure 3E**). Despite the cryo-EM structures indicating S6 lies in close proximity to CD79B (21), substitution to the bulky W or Y at position S6 is well tolerated, possibly because aromatic amino acids are generally favorable at the edges of TMDs. Mutations at T20 and T21 (to V) have been reported to be non-disruptive of normal BCR function (14), further supporting our observation that most substitutions are tolerated here.

## Discussion

With several cryo-EM structures in detergent micelles (21–23), mutagenesis in cellular plasma membranes (13–17, 39), computational simulations in model lipid bilayers (19, 20, 40, 41) and assembly and cysteine crosslinking in ER membranes (19, 20), the BCR complex now has a rich landscape of data providing complementary insights to further our understanding of the principles that govern its assembly and surface expression. The extensive dataset presented here cross-validates a number of previous mutagenesis studies and these are summarized with references in **Supplementary Table 1**. Our data report on a crucial but early step in the biological function of the BCR – successfully reaching the cell surface. The potential to perform similar assays using this library on subsequent steps such as B cell activation and antigen presentation could provide an even more complete picture of the effects of BCR mutations on B cell function.

Our data highlighted the amino acid residues in the mIg TMD that drive key interactions, and the most mutation-intolerant positions are also the most highly conserved between BCR isotypes and across species. The observation that most of these residues lie at the mIg dimer interface supports the hypothesis that formation of a stable mIg dimer is a crucial step in assembly of all BCRs, and it is consistent with previous simulations that showed all five mouse and human BCR isotypes adopt similar mIg TMD structures (19, 20). Mouse mIgD is an interesting outlier, where simulations indicated the homodimer may be less compact and more dynamic (20) than other isotypes in the N-terminal (top) half of the structure. Mouse mIgD has M at the equivalent position to mIgM A5, which is directly in the homodimer interface. This position is not highly conserved, and M is well tolerated in the context of mIgM surface expression in our screen, though more rigid branched aliphatic and aromatic amino acids are not (see **Figure 2A**). Whether these differences translate to significant structural divergence in assembled complexes is not yet known, since there are no structures of intact IgD BCRs.

We observed here that V22 is a conserved position highly sensitive to mutations, but a specific function had not previously been ascribed to it. The effects we observed likely stem from its position at the C-terminal end of the mIg dimer interface, and we note that this is akin to a similarly situated V in the T cell receptor (TCR), the BCR’s counterpart in T cells. Indeed, mutation of this V in the TCR to a bulky aromatic residue (F) attenuated assembly of the octameric complex, likely by disrupting the close packing of the central TCRαβ dimer (42, 43). This comes as an additional feature shared by these two receptors that we previously reported form extremely similar and highly conserved structures in their TMDs (19, 20) and underscores their central roles in stable assembly.

The residue W2, despite being highly conserved, is mostly tolerant of mutation to any other amino acid in our surface expression assay (**Figure 2A**). This is in agreement with previous mutagenesis work showing the mutant W2L shows no defects in surface expression or signaling, and an effect is only seen further downstream as reduced antigen presentation activity (14). We suggest that the evolutionary conservation at this position is linked not to expression or stability but instead to some other function. Two conserved leucine positions, L13 and L14, display a preference for large and/or branched aliphatics (I/L/V/M) over other energetically acceptable TMD residues, suggesting that their conservation derives from more than just the status of leucine as the most common amino acid in TMDs. The cryo-EM structures show both L13 and L14 face lipid in one protomer within the mIgM dimer, and pack loosely against I/L side chains from CD79AB in the other (22, 23). This arrangement is consistent with the weak but clear selection against small and aromatic amino acids reflected in our data and indicates that some complementary methyl packing is favorable at these interfaces.

Taken together, the data presented here illustrate the comprehensive nature of the information provided by a deep mutational scanning study of transmembrane receptor assembly and emphasize the utility of the approach for the immune receptor field specifically. These data could be useful in training computational protein structure and variant-effect predictors, which still struggle to faithfully predict TMD interactions due to the scarcity of high-resolution structural and biochemical data available for this challenging class of proteins. Additionally, we anticipate that this dataset could act as a diagnostic reference to understand the mechanism of disease for any patients identified with mutations in the mIg TMD, as we reported previously for novel oncogenic mutations identified by DMS in the human thrombopoietin receptor TMD (24). Human and mouse mIgM proteins are identical in the region covered by our scan, with the single exception of a change at T3 (to A). The only missense TMD variants identified in human exome sequencing at frequencies greater than 1/100,000 (equivalent to A5T [variant ID rs751188409], V10I [rs759494596] and V22I [rs772304630]) are well tolerated, consistent with maintenance of the rare alleles in the human population. Conversely, mutations that disrupt IgM BCR expression may be expected to have effects similar to µ heavy chain deletion, which causes an autosomal recessive form of the primary B-cell immunodeficiency agammaglobulinemia (5) though none to our knowledge have been identified in patients.

## Data availability statement

Fitness scores for the mIgM transmembrane domain DMS screen have been deposited in the Multiplexed Assays of Variant Effect Database (MaveDB; www.mavedb.org) with accession number urn:mavedb:00001202-a.

## Ethics statement

Ethical approval was not required for the studies on animals in accordance with the local legislation and institutional requirements because only commercially available established cell lines were used.

## Author contributions

SR: Conceptualization, Data curation, Formal analysis, Investigation, Methodology, Writing – original draft, Writing – review & editing. MG: Methodology, Writing – review & editing. MaC: Conceptualization, Methodology, Project administration, Supervision, Writing – review & editing. MeC: Conceptualization, Data curation, Formal analysis, Investigation, Methodology, Project administration, Software, Supervision, Writing – review & editing.
