# Supplementary material for: Deep mutational scanning reveals transmembrane features governing surface expression of the B cell antigen receptor
Source: Front Immunol. 2024 Jul 23;15:1426795. doi: 10.3389/fimmu.2024.1426795 (PMC11300204; doi:10.3389/fimmu.2024.1426795)
Supplement: Supplementary file 1 [file DataSheet_1.pdf]

Deep mutational scanning reveals transmembrane features governing surface expression of the B cell antigen receptor

Samyuktha Ramesh [1,2], Margareta Go [1,2], Matthew E. Call [1,2*†], Melissa J. Call [1,2*†]

1. *Structural Biology Division, Walter and Eliza Hall Institute of Medical Research, Melbourne, Victoria, Australia*
2. *Department of Medical Biology, University of Melbourne, Melbourne, Victoria, Australia*

* *Address correspondence to Matthew E. Call (mecall@wehi.edu.au) or Melissa J. Call (mjcall@wehi.edu.au)*

† *These authors have contributed equally to this work and share senior authorship*

Supplementary Table 1

				Previously published results				
Native residue	Mutated to	Isotype	DMS surface expression	Surface expression	BCR stability In detergent	Signaling	Antigen presentation	References
W2	L	M	+	+		+	-	[1]
	A	G/M	+		-			[2]
F8	W	G/M	-		-			[2, 3]
F12	W	G/M	-		-			[2, 3]
S15	A	M	+	+	+/-	+/-	+	[1, 4, 5]
	V	M	+		-			[5]
	L	M	+		+			[5]
	H	G/M	-		-			[2]
Y18	F	M	+	+	+/-	+/-	-	[1, 4-8]
S19	A	M	+	+	+	+	+	[1, 5, 6]
	V	M	-		-			[5]
	L	M	-		-			[5]
T20	V	M	+	+		+		[1]
T21	V	M	+	+		+		[1]
T23	V	M	+	+	+	+/-		[1, 4, 5]

Supplementary Table 1. Summary of previously published HC TMD single amino acid mutagenesis results. + : wildtype-like behavior, - : defective.

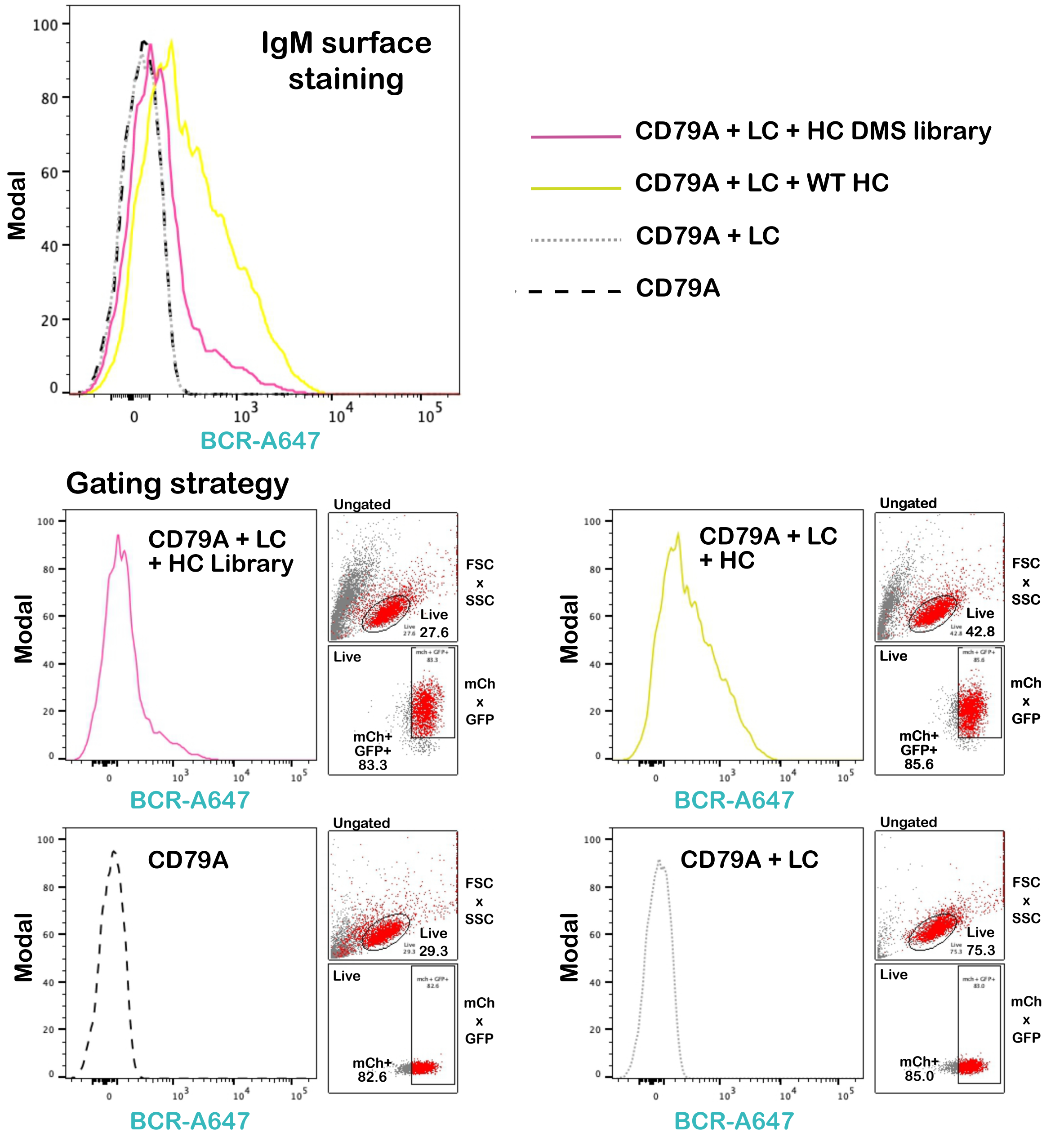

Supplementary Figure 1. Reconstitution of the IgM BCR in CD79A⁻ HC⁻ J558L cells. Surface anti-IgM staining of the mouse B cell line J558L (which lacks endogenous CD79A and HC) once reconstituted with CD79A (black dashed line), CD79A and LC (grey dotted line), CD79A, LC and wildtype HC (yellow solid line) or CD79A, LC and HC DMS library (pink solid line). The gating strategy for each sample is also shown.

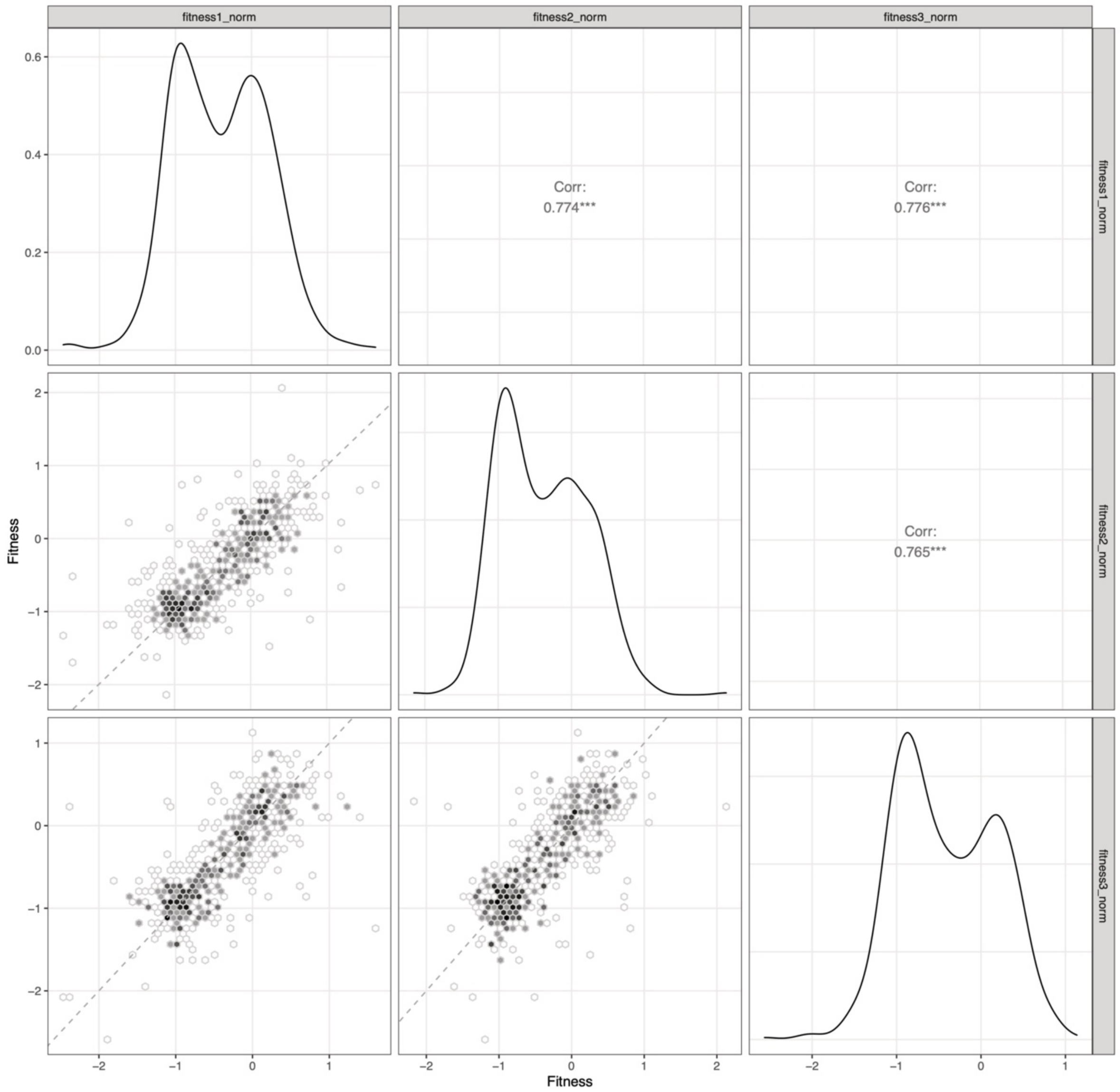

Supplementary Figure 2. Replicate fitness correlations showing agreement among the three independent viral transductions. The diagnostic plot is a scatterplot matrix generated by DiMSum [9] depicting correlations between fitness estimates from all replicates after normalisation. Matrix cells in the upper right triangle show pairwise Pearson correlation coefficients. Matrix cells in the lower left triangle show scatterplot equivalents (hexagonal heatmaps of 2d bin fitness). Matrix diagonal cells indicate fitness score densities. Only variants retained during error model fitting are included.

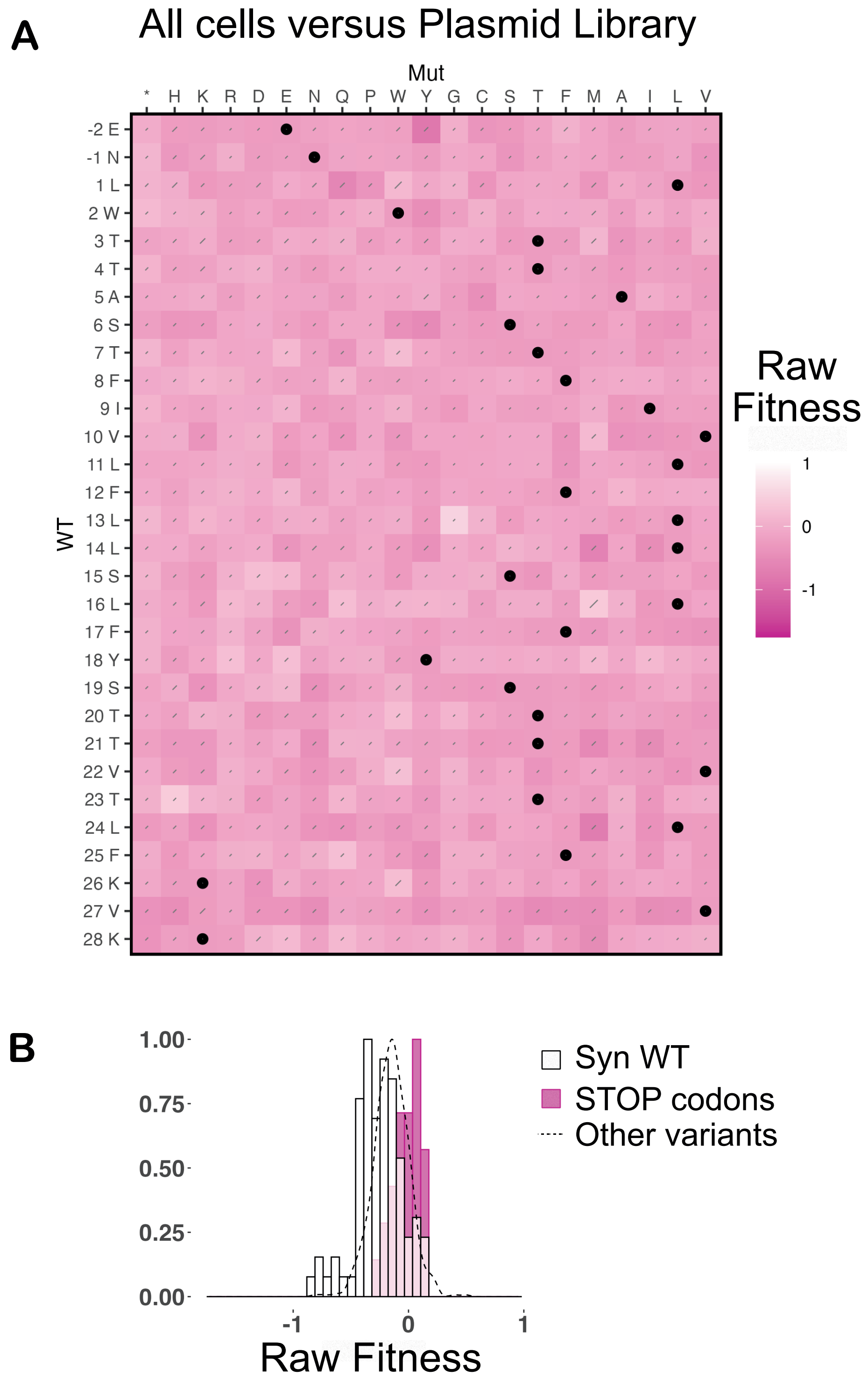

Supplementary Figure 3. Variants do not influence cell growth kinetics.
(A) Sequence-Function map of reads combined from all gates, 4-days post-transduction, compared to plasmid library. (B) Distribution of scores from Sequence-Function map.

References

1. Blum, J.H., T.L. Stevens, and A.L. DeFranco, *Role of the mu immunoglobulin heavy chain transmembrane and cytoplasmic domains in B cell antigen receptor expression and signal transduction.* J Biol Chem, 1993. **268**(36): p. 27236-45.
2. Ma, X., et al., *Cryo-EM structures of two human B cell receptor isotypes.* Science, 2022. **377**(6608): p. 880-885.
3. Su, Q., et al., *Cryo-EM structure of the human IgM B cell receptor.* Science, 2022. **377**(6608): p. 875-880.
4. Pleiman, C.M., N.C. Chien, and J.C. Cambier, *Point mutations define a mIgM transmembrane region motif that determines intersubunit signal transduction in the antigen receptor.* J Immunol, 1994. **152**(6): p. 2837-44.
5. Ramesh, S., et al., *T cell and B cell antigen receptors share a conserved core transmembrane structure.* Proc Natl Acad Sci U S A, 2022. **119**(48): p. e2208058119.
6. Shaw, A.C., et al., *Mutations of immunoglobulin transmembrane and cytoplasmic domains: effects on intracellular signaling and antigen presentation.* Cell, 1990. **63**(2): p. 381-92.
7. Grupp, S.A., et al., *Signaling-defective mutants of the B lymphocyte antigen receptor fail to associate with Ig-alpha and Ig-beta/gamma.* J Biol Chem, 1993. **268**(34): p. 25776-9.
8. Mitchell, R.N., et al., *Intracellular targeting of antigens internalized by membrane immunoglobulin in B lymphocytes.* J Exp Med, 1995. **181**(5): p. 1705-14.
9. Faure, A.J., et al., *DiMSum: an error model and pipeline for analyzing deep mutational scanning data and diagnosing common experimental pathologies.* Genome Biology, 2020. **21**(1): p. 207.
